# Long-term efficacy data for the recombinant zoster vaccine: impact on public health and cost effectiveness in Germany

**DOI:** 10.1080/21645515.2021.2002085

**Published:** 2021-12-14

**Authors:** Desmond Curran, Desirée Van Oorschot, Sean Matthews, Johannes Hain, Ahmed Ehab Salem, Magdalena Schwarz

**Affiliations:** a Value Evidence, GSK, Wavre, Belgium; b Value Evidence Freelance c/o GSK, Wavre, Belgium; c Vaccines, GSK, München, Germany

**Keywords:** Herpes zoster, public health impact, cost effectiveness, vaccination, number needed to vaccinate

## Abstract

The aim of the study was to update previously published public health impact and cost-effectiveness analyses of the recombinant zoster vaccine (RZV), in the German population aged ≥50 years of age (YOA), with the latest vaccine efficacy (VE) estimates against herpes zoster (HZ). The updated estimates are derived from a long-term follow-up study. A previously published multi-cohort Markov model following age cohorts over their lifetime was used. Demographic, epidemiological, cost, and utility data were based on German specific sources. Vaccine coverage was assumed to be 40%, with a second dose compliance of 70%. The estimated VE at time 0 was 98.9% (95% C.I.: 94.0–100%) with an annual waning of 1.5% (95% CI: 0.0–3.4%) for the age group 50–69 YOA. Corresponding values were 95.4% (95% C.I.: 89.7–100%) and 2.3% (95% CI: 0.3–4.4%) for the age group ≥70 YOA. It was estimated that, over the remaining lifetime since vaccination, RZV would prevent approximately 884 thousand (K), 603 K, and 538 K HZ cases in three age cohorts 50–59, 60–69, and ≥70 YOA, respectively. The number needed to vaccinate to prevent one HZ and one postherpetic neuralgia case was 6 and 36 (50–59 YOA cohort), 6 and 34 (60–69 YOA cohort), 10 and 48 (≥70 YOA cohort). The incremental cost-effectiveness ratio of vaccination ranged from €26 K/quality-adjusted life year (QALY) in 60 YOA to €35 K/QALY in 70 YOA. Due to the higher, sustained, RZV VE, improved public health and cost-effectiveness results were observed compared to previous analyses.

## PLAIN LANGUAGE SUMMARY

What is the context?

- Shingles is a viral infection caused by the reactivation of the chickenpox virus. It causes a painful rash that lasts for several weeks.
- The incidence and severity of shingles increase with age. In Germany alone there are approximately 400,000 new cases annually.
- Vaccination can help prevent shingles.
- Previous studies, based on data collected up to four years post-vaccination, estimated the number of shingles cases prevented.

What is new?

- Here, we use data from the same studies followed over a longer-term to update previous analyses in the German population.
- We found, based on data up to 8 years following vaccination, that:

○ In adults 50-69 years: the vaccine initially prevents 98.9% of cases, with a reduction of 1.5% each year(for example, after one year, it would prevent 97.4% of cases).

○ In adults over 70 years of age: the vaccine initially prevents 95.4% of cases, with a reduction of 2.3% each year (for example, after one year, it would prevent 93.1% of cases).

○ Vaccination would reduce the number of shingles cases by 0.9 million in a cohort of adults aged 50-59 years, 0.6 million in adults 60-69 years, and 0.5 million in adults older than 70 years, over the remainder of their lifetime.

What is the impact?

- The study provides more certainty regarding results as it is based on the most complete/up to date data.
- The results showed the potential of Shingrix to prevent shingles while at the same time providing good value for money.

## Introduction

Herpes zoster (HZ) is a painful and debilitating condition caused by a reactivation of varicella-zoster virus (VZV), which as a primary infection causes chickenpox.^1^ The incidence and severity of HZ are known to increase markedly with age associated with an age-related decline in immunity. The lifetime risk of developing HZ is estimated at approximately 30%.^2^ Pain that continues after the rash has healed is termed postherpetic neuralgia (PHN, often defined as pain persisting or appearing 90 days after rash onset), a chronic neuropathic pain syndrome.^3^ HZ burden on the health-care system in Germany is substantial with over 400 thousand (K) HZ cases annually resulting in a total cost to society of approximately €182 million (M).^4,5^

A zoster vaccine live (ZVL, *Zostavax*, Merck Sharp & Dohme Corp), was licensed by the European Medicines Agency (EMA) in 2006. However, the German National Immunization Technical Advisory Group (NITAG) Standing Committee on Vaccination (STIKO) decided in 2017 against recommending a standard HZ vaccination with ZVL.^6^ An adjuvanted recombinant zoster vaccine (RZV; *Shingrix*, GSK) was developed to prevent HZ. It is a two-dose vaccine regime combining recombinant VZV glycoprotein E and the AS01_B_ adjuvant system.^7^ Two multinational phase III randomized, observer-blinded, placebo-controlled clinical trials were conducted concurrently at the same study sites using the same methods to assess the efficacy of RZV in preventing HZ in two adult populations. The ZOE-50 study (NCT01165177) included patients aged 50 years and older^8^ and ZOE-70 study (NCT01165229) included patients aged 70 years and older,^9^ with efficacy estimates presented up to 4 years. A long-term follow-up study (ZOE-LTFU), i.e. extension of the original study populations, is ongoing. An interim analysis of the ZOE-LTFU study presenting the vaccine efficacy (VE) estimates out to 8 years post-initial vaccination was recently published.^10^

In 2017, we published an assessment of the potential public health impact of HZ vaccination in Germany, based on the then available short-term efficacy data as reported in the ZOE-50 and ZOE-70 studies.^11^ This was followed-up by an associated cost-effectiveness analysis in the German population aged ≥60 and ≥50 years old.^12,13^ It was estimated that, over the remaining lifetime since vaccination, RZV would reduce the number of HZ cases by approximately 1.7 million, assuming a vaccine coverage rate of 40% in adults ≥50 years of age (YOA).^11^ The incremental cost-effectiveness ratio (ICER) of vaccination was approximately €35 K, €37 K, and €44 K/quality-adjusted life year (QALY), for the age cohorts ≥50, ≥60, and ≥70 YOA, respectively.^12,13^ In 2018, STIKO recommended RZV: (1) for all people 60 YOA and over (standard vaccination); (2) people from 50 YOA who have an elevated risk of HZ and PHN owing to increased health risks as a consequence of an underlying disease or immunosuppression (indication-based vaccination).^14^

VE and duration of protection are important factors that policymakers consider when developing HZ vaccination policy recommendations and for reimbursement.^6,14^ The scope of the current study is to: (1) estimate vaccine efficacy at time 0 and subsequent waning rates of the RZV vaccine based on the ZOE-LTFU clinical trial data with VE estimates out to 8 years following initial vaccination; (2) update the public health impact analysis and cost-effectiveness analysis of the RZV vaccination in Germany; (3) compare the updated results with the previous results; and (4) explore the consequences for the STIKO recommendation from 2018.^6^

## Methods

An interim analysis of ZOE-LTFU, following up the original ZOE study populations, presented the VE estimates out to 8 years following initial vaccination.^10^ Details of the study design and methodology are provided in Boutry et al.^10^ We used the observed ZOE-LTFU clinical trial VE analysis by year separated into the age groups 50–69 and ≥70 YOA (see supplementary Table S1) to estimate the VE at time 0 (i.e. take) and slope (annual waning) of efficacy over time using a linear regression model. A bootstrap analysis was used to estimate 95% confidence intervals (C.I.) around the estimates for VE take and annual waning.^15^ The bootstrap analysis used the summary information reported in the clinical trial (e.g. treatment group, year, sample size, number of HZ cases, follow-up time) to generate samples representing the original study sample (size N). Sampling was carried out with replacement (size N). Thus, some “subjects” in the original sample were included several times, while others were excluded altogether. A linear regression analysis was then fitted on this sample generating a point estimate for the VE take and waning. One thousand simulations were performed, with the 95% C.I. obtained by sorting the data and taking the 25th and 975th observations, respectively.

ZOster ecoNomic Analysis (ZONA) is a static multi-cohort Markov model developed in MS Excel.^11,13^ Cohorts are split into 5 age groups for people ≥50 YOA (i.e. 50–59, 60–64, 65–69, 70–79, ≥ 80). The model follows all subjects within a cohort over their remaining lifetime from the year of vaccination with annual cycle lengths. As such, all subjects remain in their initial cohort and all subsequent events are counted in that cohort only. Supplementary Figure S1 provides an overview of the model structure. Two different HZ vaccination strategies were compared in this analysis; no vaccination (control), and vaccination with RZV.

For the current study, we used the updated efficacy/waning estimates to update previously published analysis: (1) public health impact analysis focusing on three age cohorts: 50–59, 60–69, and ≥70 YOA,^11^ (2) cost-effectiveness analysis of vaccination at ages 50, 60, 65 and 70 YOA, and ≥50, ≥60, and ≥70 YOA age groups.^13^ The age cohorts were selected in order to capture age-dependent differences in disease incidence, complications, outcomes, costs, and potential public health decision making.

### Model inputs

For the base-case analysis, all model inputs, excluding vaccine characteristics, including demographics, epidemiology, costs and utility values used German specific data, and remain consistent with the assumptions used in previous publications.^11,13^ Coverage of the first dose was assumed to be 40% and compliance of the second dose of RZV is assumed to be 70% in the base-case. Further details regarding the model structure, model inputs and assumptions are provided in the supplementary text and elsewhere.^11,13^

For the public health impact analysis, the model cohort sizes reflected the size of the German population for each age cohort, i.e. including approximately 13 M, 9.5 M, and 13 M individuals in the three age cohorts 50–59, 60–69, and ≥70 respectively. We estimated the number needed to vaccinate (NNV) to prevent one HZ case and to prevent one PHN case, respectively. Two alternative scenarios were evaluated: (1) assuming that all vaccinated individual received one dose of RZV only (i.e. 0% 2nd dose compliance) and (2) all vaccinated individual received two doses (i.e. 100% 2nd dose compliance). The number of hospitalizations and general practitioner visits avoided were calculated for each vaccine scenario.

For the cost-effectiveness analysis, the model follows hypothetical cohorts of 1 million people, to ensure that comparisons on the cost outcomes can be made between age cohorts. To allow direct comparisons with our previous cost-effectiveness results, a price of €110 per dose was assumed, reflecting the price to retailer reduced by the obligatory pharmacy and manufacturer rebates, corresponding to a price to wholesaler of €84.5 per dose.^13^ Since there is no willingness to pay (WTP) threshold to define cost-effectiveness in Germany, a hypothetical threshold of €50,000/QALY, which is commonly used in other economic evaluations relevant to Germany, was used.^13,16,17^

A deterministic sensitivity analysis (DSA) was performed for the cost-effectiveness analysis of the ≥60 age cohort. A probabilistic sensitivity analysis (PSA) was performed using 1,000 Monte-Carlo simulations for both the ≥50 and ≥60 age cohorts. A Beta distribution was assumed for VE and waning parameters using the upper bound and lower bound values, obtained from the bootstrap 95% confidence intervals estimate, to calculate standard errors. Ranges for the other parameters included in the DSA and PSA are presented in supplementary Table S2.

An additional scenario analysis was conducted by updating both direct and indirect costs of HZ to 2020 values. The ICER was estimated for different price points using both the updated efficacy and cost data.

## Results

### Vaccine efficacy and waning

**Figure 1.**
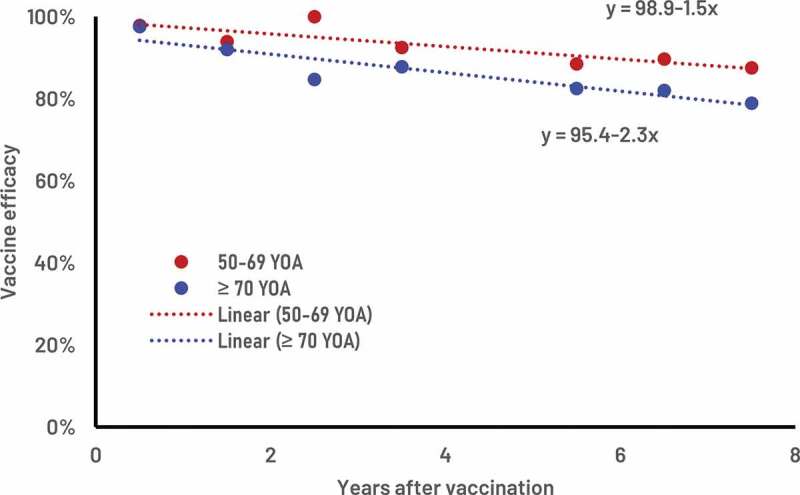
ZOE-LTFU clinical trial, recombinant zoster vaccine efficacy estimates (red and blue dots) and the corresponding estimates of vaccine efficacy and waning over time.

ZOE-LTFU: zoster long-term follow-up study; YOA: years of age.

Supplementary Table S1 provides the ZOE-LTFU clinical trial estimates of RZV VE values over time for the two age groups 50–69 and ≥70 YOA. Figure 1 presents the corresponding clinical trial values and the fitted estimates of the VE and waning rates over time. The estimated VE at time 0 was 98.9% (bootstrap 95% C.I. 94.0% – 100%) with an associated annual waning of 1.5% (bootstrap 95% C.I. 0.0% – 3.4%) for the age group 50–69 YOA. The estimated VE at time 0 was 95.4% (bootstrap 95% C.I. 89.7% – 100%) with an annual waning of 2.3% (bootstrap 95% C.I. 0.3% – 4.4%) for the age group ≥70 YOA.

### Public health impact

**Figure 2.**
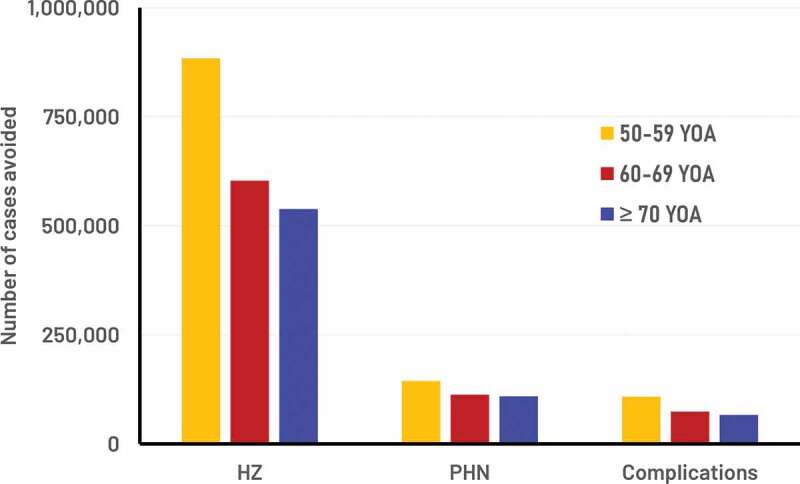
Number of cases avoided with RZV from the year of vaccination over the remaining lifetime by age cohort.

**Figure 3.**
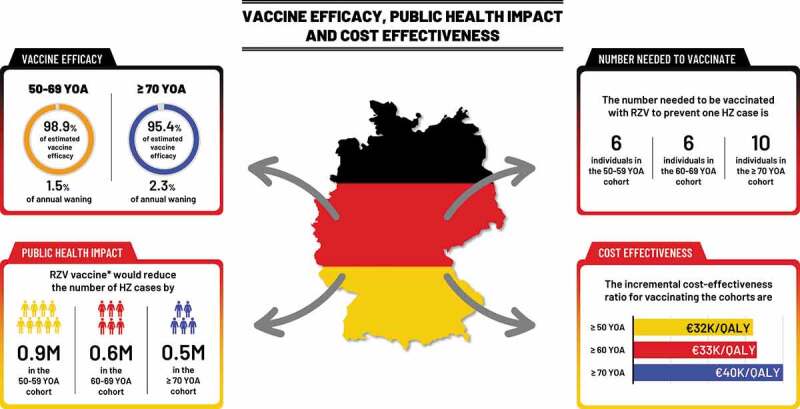
RZV vaccine efficacy estimates with associated annual wanings, public health impact in the German population and cost-effectiveness.

The model cohort sizes reflected the German population, i.e. including approximately 13 M, 9.5 M, and 13 M individuals in the three age cohorts 50–59, 60–69, and ≥70 respectively. Figure 2 presents the public health impact over the remaining lifetime with RZV assuming a coverage rate of 40%, and a second dose compliance of 70%. It was estimated that the RZV vaccine would reduce the number of HZ cases by approximately 2 M overall (i.e. 884 thousand (K), 603 K and 538 K HZ in the three age cohorts 50–59, 60–69, and ≥70, respectively (see Figure 3)). Table 1 presents the results for the base-case, and also assuming that all vaccinated individual received one dose of RZV only (i.e. 0% 2nd dose compliance) and alternatively that all vaccinated individual received two doses (i.e. 100% 2nd dose compliance). The table shows that two doses of RZV is expected to prevent 2.8 times as many cases of HZ as compared to one dose.

**Table 1.** Public health impact of both RZV and ZVL under base-case assumptions of 40% coverage (RZV second dose compliance of 70%) over a lifetime horizon from the age of vaccination

	Cases avoided with RZV			
	2nd dose compliance 0%	2nd dose compliance 70%	2nd dose compliance 100%	Ratio of 2 vs 1 doses
HZ	883,484	2,025,787	2,515,346	2.8
PHN	150,004	365,393	457,703	3.1
Complications	108,227	248,159	308,130	2.8
Deaths	120	542	724	6.0
Hospitalization	38,887	104,586	132,742	3.4
GP visits	4,592,284	11,154,298	11,475,288	2.5

GP: general practitioner; HZ: herpes zoster; PHN: postherpetic neuralgia; RZV: recombinant zoster vaccine; ZVL: zoster vaccine live.

*In vaccinated subjects compared to no vaccination over the lifetime of the respective cohorts.

Approximate cohort sizes: 13 million, 9.5 million and 13 million individuals in 50–59, 60–69, and ≥70 YOA, respectively. HZ: herpes zoster; PHN: postherpetic neuralgia; RZV: recombinant zoster vaccine; YOA: years of age. Complications: complications other than PHN.

*A fixed cohort of a total of 1 M subjects at each age is assumed, of which 40% were assumed to be vaccinated with the first dose and 70% would receive a second dose of RZV. HZ: herpes zoster; K: thousands; M: million; PHN: postherpetic neuralgia; QALY: quality adjusted life-years, RZV: recombinant zoster vaccine; YOA: years of age.

Table 2 presents the number needed to vaccinate (NNV) to prevent one HZ case and to prevent one PHN case, respectively. In the base-case 6, 6 and 10 individuals need to be vaccinated with RZV to prevent one HZ case in the three cohorts aged 50–59, 60–69, and ≥70, respectively (see Figure 3). The corresponding NNV for PHN are 36, 34, and 48, in the three age cohorts, respectively. When 2 doses of RZV are given to all subjects the NNVs to prevent one HZ case in the three age cohorts are 5, 5, and 8 compared with 17, 12, and 20 when only one dose of RZV is administered.

**Table 2.** Number needed to vaccinate to prevent one HZ case and one PHN case under base-case assumptions of 40% coverage (RZV second dose compliance of 70%) over a lifetime horizon from the age of vaccination

	HZ			PHN		
Second dose compliance	0%	70%	100%	0%	70%	100%
50–59 YOA	17	6	5	122	36	28
60–69 YOA	12	6	5	69	34	28
≥ 70 YOA	20	10	8	99	48	39

HZ: herpes zoster; PHN: postherpetic neuralgia; RZV: recombinant zoster vaccine; YOA: years of age. Note estimated number needed to vaccinate values were rounded up to the nearest integer.

### Cost-effectiveness

Table 3 presents the results of the cost-effectiveness analysis of RZV assuming a fixed cohort of a total of 1 M subjects at each age (i.e. at 50, 60, 65, and 70 YOA). Vaccination costs would be around €81 M per cohort, yielding an ICER of about €26 K per QALY gained in 60 YOA to €35 K/QALY in 70 YOA. The cost-effectiveness results of vaccinating age cohorts of 1 M people ≥50, ≥60, and ≥70 YOA are presented in Table 4. The ICER for vaccinating the cohort aged ≥50 was approximately €32 K/QALY, compared with €33 K/QALY for vaccinating the ≥60 cohort and 40 K/QALY for vaccinating the ≥70 cohort (see Figure 3).

**Table 3.** Cost-effectiveness of RZV vs no vaccination for various vaccination starting ages: assuming a fixed cohort of 1 million with a vaccine coverage of 40%

Age	50 YOA	60 YOA	65 YOA	70 YOA
HZ cases avoided	68,042	65,410	60,735	47,594
PHN cases avoided	11,087	11,919	11,700	9,568
Total costs (discounted)	53,716,953	54,325,317	56,876,134	64,859,931
QALYs gained (discounted)	1,818	2,127	2,178	1,871
ICER	€29,547/ QALY	€25,536/ QALY	€26,116/ QALY	€34,663/ QALY

HZ: herpes zoster; PHN: postherpetic neuralgia; RZV: recombinant zoster vaccine; YOA: years of age; QALY: quality-adjusted life year; ICER: incremental cost-effectiveness ratio.

**Table 4.** Cost-effectiveness of RZV vs no vaccination for various age cohorts

Age cohort	≥ 50 YOA	≥ 60 YOA	≥ 70 YOA
HZ cases avoided	57,071	50,737	41,511
PHN cases avoided	10,294	9,836	8,379
Complications other than PHN avoided	6,991	6,215	5,085
HZ-related deaths avoided	15	19	22
Discounted life-years gained	67	87	102
Discounted QALYs gained	1,858	1,881	1,683
Total costs (discounted)	58,959,402	61,986,534	66,766,789
ICER	€31,735/QALY	€32,956/QALY	€39,676/QALY
Direct and indirect costs			
updated to 2020 values			
Total costs (discounted)	€57,054,928	€60,449,516	€65,810,032
ICER	€30,710/QALY	€32,139/QALY	€39,107/QALY
Vaccine Price			
€133.62/dose			
Total costs (discounted)	€73,084,026	€76,478,615	€81,839,131
ICER	€39,337/ QALY	€40,661/ QALY	€48,632/ QALY

HZ: herpes zoster; PHN: postherpetic neuralgia; RZV: recombinant zoster vaccine; YOA: years of age; QALY: quality-adjusted life year; ICER: incremental cost-effectiveness ratio.

The 2021 price of RZV is €133.62 per dose (i.e. average price to be paid by payers for one dose across all 17 health care regions via office supply) corresponding to a price to wholesaler (PTW) of €106.18 per dose.

The results of the deterministic sensitivity analyses (DSA) are given in supplementary Figure S3 (cohort ≥60 YOA). Incidence of HZ and probability of subsequently developing PHN showed the largest variation around the ICER. The cost-effectiveness acceptability curve indicated that for 94.0% and 92.9% of all simulations in the cohorts ≥50 and ≥60 YOA, respectively, the ICER was below the hypothetical WTP threshold of €50,000/QALY (i.e. representing a cost-effective intervention).

Table 4 includes a presentation of the ICER values when direct costs and indirect costs of HZ and PHN were updated to 2020 values. Note there was a small decrease in the ICERs when the updated costs were applied. The economically justifiable price, i.e. reflecting the maximum price resulting in the ICER being equal to the hypothetical WTP threshold of €50,000/QALY, was €163, €160, and €137 per dose for the 3 age cohorts ≥50, ≥60, and ≥70 YOA, respectively.

## Discussion

Initial data available on efficacy of the RZV vaccine was limited to a maximum of 4 years post vaccination.^8,9,11^ Although only modest waning of vaccine-induced protection was observed,^18^ the lack of data beyond 4 years was documented as a limitation of several cost-effectiveness publications.^19–21^ The primary clinical trial results from the interim analysis of the ZOE-LTFU study suggests that the VE estimates at 8 years post vaccination were 84.3% overall.^10^ In this manuscript, we have provided more granular data from that study demonstrating that VE estimates at 8 years post vaccination were 87.5% in individuals aged 50–69 and 78.9% in individuals aged ≥70 YOA (see supplementary Table S1). Szucs et al., in a review of HZ vaccine cost-effectiveness manuscripts noted that a limitation of most modeling studies was that outdated input data were used.^22^ The authors noted that cost-effectiveness models should be updated when new evidence comes available to support the effect on a potential vaccination recommendation.

In this analysis, we demonstrated based on the ZOE-LTFU clinical trial data, that the VE of RZV waned at an annual rate of 1.5% during 8 years post-vaccination in individuals aged 50–69 and at an annual rate of 2.3% in individuals aged ≥70. Using a bootstrap analysis, ranges around estimates of efficacy and waning were provided. Ranges are particularly relevant for sensitivity analysis of economic analyses.^23^ With 8 years follow-up, the ZOE-LTFU study demonstrated more robust long-term efficacy compared to the original ZOE studies and therefore provides more certainty regarding (1) durability of protection of RZV and (2) outcomes of economic models. The latter is particularly evident where 92.9% of simulations in this study were cost-effective, for the ≥60 YOA cohort, using a WTP threshold of €50,000/QALY, compared to 84% of simulations in the corresponding analysis performed in 2018.^13^

Model assumptions around efficacy and waning used in this study are further supported by long-term immunological data suggesting that there was no significant decrease in observed immune response for RZV from year 5 to year 10.^24^ Mathematical models on data up to 10 years indicate that immune responses will remain above pre-vaccination levels ≥20 years after initial vaccination.^24^

One of the aims of STIKO is to reduce the burden of HZ including complications and long-term consequences caused by HZ in older adults by vaccination.^14^ The authors noted that for ZVL, to protect individuals at the age at which the risk of disease is greatest, the individual must be vaccinated as late as possible in life. However, they also noted the limitation of that strategy, given that for ZVL the vaccine has low efficacy in older age groups. For the RZV vaccine, the projected efficacy estimates, based on the observed ZOE-LTFU clinical data, suggest that individuals vaccinated at age 50 with 2-doses would continue to have a VE against HZ of approximately 70% at 70 YOA (see supplementary Figure S2). In our model, for RZV, we did not include the potential for a reduction of PHN beyond that afforded by the reduction in HZ (i.e. on-top efficacy). In the original ZOE studies in older adults, RZV not only prevented HZ but mitigated pain associated with breakthrough HZ, resulting in less severe pain and a lower average pain.^25,26^ In the ZOE-HSCT study carried out in autologous hematopoietic stem-cell transplantation (HSCT) recipients, VE in preventing HZ was 68.2%, while the VE in preventing PHN was 89.3%, and VE in reducing the burden of illness associated with HZ-related pain was 82.5%.^27,28^ It is likely that vaccine-induced VZV-specific CD4+ T cells play a role in the attenuation of severity of breakthrough cases for both ZVL and for RZV.^26^

In the present study, we have demonstrated that vaccine efficacy and waning values for RZV provide more certainty around long-term protection. These new estimates also support that earlier vaccination against HZ provides better outcomes than previously observed. For example, the size of the German population in the age-cohorts 50–59, and ≥70 YOA were similar, e.g. approximately 13 M, respectively. The greatest benefit in terms of preventing HZ and PHN cases was observed when vaccinating the 50–59 YOA cohort compared to the ≥70 YOA cohort.

This result may appear counterintuitive, i.e. since both incidence of HZ and the probability of developing PHN increase with age. This result is mainly due to two factors, i.e. the longer life expectancy of the 50–59 YOA cohort and the long duration of protection of the RZV vaccine.

In this study, it was estimated for the base-case that six individuals would need to be vaccinated with RZV to prevent 1 HZ case for both the 50–59 and 60–69 age cohorts. The corresponding NNV for PHN were 36 and 34 for the two age cohorts, respectively. A second dose completion rate of 70% was assumed for the base-case. Real-world data suggests that the second-dose completion rates may be higher.^29^ When two doses of RZV are given to all subjects the NNVs are 5 to prevent 1 HZ case and 28 to prevent 1 PHN case for both the 50–59, and 60–69 age cohorts, respectively. These results would suggest that vaccination of the cohort of individuals aged 50–59 would show similar benefit to vaccinating the cohort aged 60–69 YOA, when 2nd dose compliance rates are close to 100%. These results are not surprising given that at year 8 post-vaccination of the ZOE-LTFU, the VE, for individuals who received both doses of RZV, in 50–69 YOAs was estimated to be 87.5%, with projected estimates at year 10 and 20 post-vaccination of approximately 85% and 70%, respectively for an individual vaccinated at age 50. It is also important to consider that in Germany annually approximately 27% of HZ cases in individuals ≥50 YOA occur in individuals aged 50–59 (see Supplemental Table S3).

Both the results of the public health and cost-effectiveness outcomes were improved by using the updated efficacy and waning estimates compared to our previously published work for Germany.^11–13^ For example, by updating the efficacy and waning values only, the ICER decreased from approximately €37 K/QALY to €33 K/QALY for the age cohort ≥60. Similarly, the ICER decreased from approximately €35 K/QALY to €32 K/QALY for the age cohort ≥50, also suggesting that, in Germany, vaccinating the population aged ≥50 is even more cost-effective than vaccinating the population aged ≥60 (i.e. the current STIKO recommendation).^11,14^

One limitation of our model is that estimates of VE waning rates, generated from clinical trials where follow-up was limited to 8 years, were used to project future waning rates. As such, there is uncertainty regarding waning rates beyond 8 years. This limitation was mitigated through scenario analysis and PSA covering the range of efficacy/waning estimates that could be expected.

## Conclusions

With 8 years data post-vaccination, the ZOE-LTFU study provides updated estimates of initial efficacy and waning of RZV. The results demonstrated that the long-term efficacy was robust in both adults 50–69 YOA and adults ≥70 YOA which provides certainty regarding the durability of protection of RZV and the outcomes of economic models. This evidence may help clinicians, payers and policymakers in their assessment of the value of RZV vaccination against HZ, not only in Germany but also in other countries where there is an unmet need regarding the prevention of HZ disease.

## Supplementary Material

Supplemental Material

## Trademark

Shingrix is a trademark owned by or licensed to the GSK group of companies.

Zostavax is a trademark owned by or licensed to Merck Sharp & Dohme Corp.
