# Supplementary material for: Long-term efficacy data for the recombinant zoster vaccine: impact on public health and cost effectiveness in Germany
Source: Hum Vaccin Immunother. 2021 Dec 14;17(12):5296–303. doi: 10.1080/21645515.2021.2002085 (PMC8904019; doi:10.1080/21645515.2021.2002085)
Supplement: Supplemental Material [file KHVI_A_2002085_SM8835.docx]

Supplementary Material

PHI and CE of RZV vaccination

in Germany

Table S1: ZOE-LTFU Clinical Study, RZV vaccine efficacy results over time by vaccine group and age group

| Age group (YOA) |  | RZV | | | | Placebo | | | | Vaccine  efficacy |
| --- | --- | --- | --- | --- | --- | --- | --- | --- | --- | --- |
|  | Year | N | HZ cases | T(year) | Incidence | N | HZ cases | T(year) | Incidence |  |
| 50-69 | 0.5 | 5631 | 1 | 5588.2 | 0.0002 | 5689 | 47 | 5617.1 | 0.0084 | 97.9% |
|  | 1.5 | 5530 | 3 | 5498.8 | 0.0005 | 5540 | 49 | 5472.0 | 0.0090 | 93.9% |
|  | 2.5 | 5449 | 0 | 5404.0 | 0.0000 | 5413 | 58 | 5345.6 | 0.0109 | 100.0% |
|  | 3.5 | 5331 | 3 | 5906.4 | 0.0005 | 5250 | 39 | 5777.9 | 0.0067 | 92.5% |
|  | 5.5 | 3292 | 3 | 3271.8 | 0.0009 | 3292 | 26 | 3271.8 | 0.0079 | 88.5% |
|  | 6.5 | 3246 | 3 | 3226.9 | 0.0009 | 3246 | 29 | 3226.9 | 0.0090 | 89.7% |
|  | 7.5 | 3202 | 3 | 2282.5 | 0.0013 | 3202 | 24 | 2282.5 | 0.0105 | 87.5% |
| ≥70 | 0.5 | 8250 | 2 | 8156.2 | 0.0002 | 8346 | 83 | 8206.2 | 0.0101 | 97.6% |
|  | 1.5 | 8039 | 7 | 7916.9 | 0.0009 | 8024 | 87 | 7860.5 | 0.0111 | 92.0% |
|  | 2.5 | 7736 | 9 | 7612.2 | 0.0012 | 7661 | 58 | 7488.4 | 0.0077 | 84.7% |
|  | 3.5 | 7426 | 7 | 7040.3 | 0.0010 | 7267 | 56 | 6859.6 | 0.0082 | 87.8% |
|  | 5.5 | 3985 | 7 | 3937.0 | 0.0018 | 3985 | 40 | 3937.0 | 0.0102 | 82.5% |
|  | 6.5 | 3851 | 7 | 3766.2 | 0.0019 | 3851 | 39 | 3766.2 | 0.0104 | 82.1% |
|  | 7.5 | 3674 | 4 | 2877.6 | 0.0014 | 3674 | 19 | 2877.6 | 0.0066 | 78.9% |

N: number of subjects; HZ: herpes zoster; T: follow-up time; RZV: recombinant zoster vaccine; YOA: years of age.

## Figure S1: Model structure


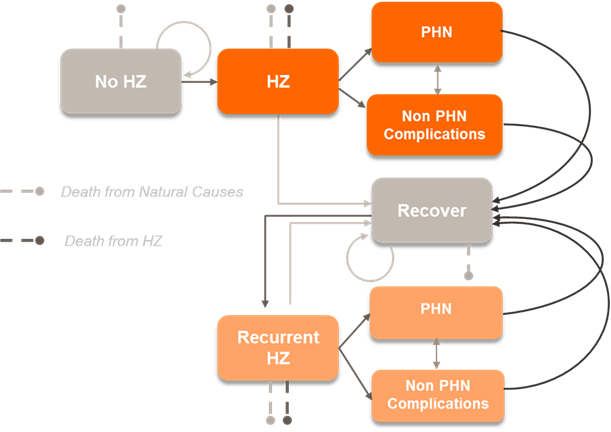


Reproduced from Curran et al. ^11^

HZ: herpes zoster; PHN: postherpetic neuralgia.

Table S2: Input values for base-case and sensitivity analyses. Reproduced from Van Oorschot et al.^13^

| ***Parameter*** | ***Age group (YOA)*** | ***Base-case value*** | ***DSA (Min.)*** | ***DSA (Max.)*** |
| --- | --- | --- | --- | --- |
| ***Epidemiology*** | | | | |
| Population size** | 60-64  65-69  70-79  ≥80 | 5,202,056  4,331,884  8,239,091  4,729,203 | N/A | N/A |
| HZ – Incidence and recurrence | 60-64  65-69  70-79  ≥80 | 0.0100  0.0114  0.0134  0.0139 | 0.0063  0.0072  0.0085  0.0094 | 0.0103  0.0118  0.0138  0.0148 |
| PHN – Probability of HZ Cases | 60-64  65-69  70-79  ≥80 | 15.4%  17.5%  20.0%  20.4% | 5.10%  5.10%  6.78%  11.17% | 20.51%  20.51%  24.05%  26.03% |
| HZ-related fatality | 60-64  65-69  70-74  75-79  80-84  ≥85 | 0.003%  0.005%  0.010%  0.025%  0.043%  0.165% | 0.000%  0.002%  0.004%  0.011%  0.022%  0.095% | 0.013%  0.016%  0.024%  0.046%  0.076%  0.263% |
| ***Quality of Life*** | | | | |
| Baseline Utilities | 60-64  65-69  70-79  ≥80 | 0.975  0.976  0.959  0.895 | N/A | N/A |
| Disutilities HZ only | 60-69  ≥70 | 0.018  0.019 | -30% | +30% |
| Disutilities HZ and PHN | ≥50 | 0.158 | -30% | +30% |
| ***Costs*** | | | | |
| Direct medical - per HZ case | 60-64  65-69  70-79  ≥80 | €226  €226  €203  €320 | €179  €179  €159  €249 | €270  €270  €252  €394 |
| Direct medical - per PHN case | 60-64  65-69  70-79  ≥80 | €1,349  €1,349  €1,172  €642 | €714  €714  €717  €251 | €2,125  €2,125  €1,785  €1,157 |
| Indirect – per HZ case | 60-64  65-69  70-79  ≥80 | €112  €112  €11  €11 | -20% | +20% |
| Indirect – per PHN case | 60-64  65-69  70-79  ≥80 | €788  €788  €46  €34 | -20% | +20% |
| ***Vaccine costs*** | | | | |
| Price per dose | All | €110 | €100 | €120 |
| Administration costs per dose | All | €7.55 | €6.30 | €9.43 |
| Adverse Events total† | 60-64  65-69  70-79  ≥80 | €1.86  €1.86  €1.81  €1.85 | -50% | +100% |
| ***Discounting*** | | | | |
| Costs | All | 3% | 1% | 5% |
| Outcomes | All | 3% | 1% | 5% |

DSA: Deterministic sensitivity analysis; HZ: Herpes zoster; PHN: Postherpetic neuralgia.

RZV vaccine efficacy over time by vaccine and age group

Based on the ZOE-LTFU clinical trial data (see Figure 1), it was assumed that for subjects aged 50-69 YOA, the HZ efficacy for 2-doses of RZV wanes at 1.5% until the age of 69 years and at 2.3% for all subjects aged ≥70 YOA (See Figure S1).

Figure S2: RZV 2-dose HZ vaccine efficacy waning assumptions based on the efficacy and waning estimates over time for three different age groups


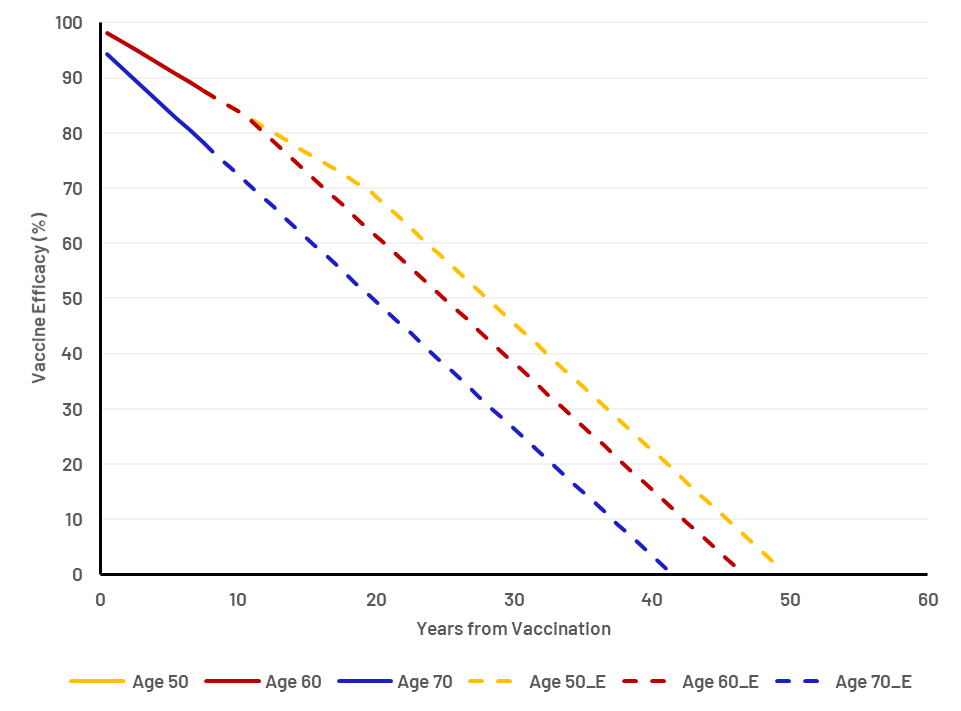


E: extrapolated, HZ: herpes zoster; RZV: recombinant zoster vaccine; YOA: years of age.

Figure S3: DSA top-10 results for the cohort aged ≥ 60 YOA.


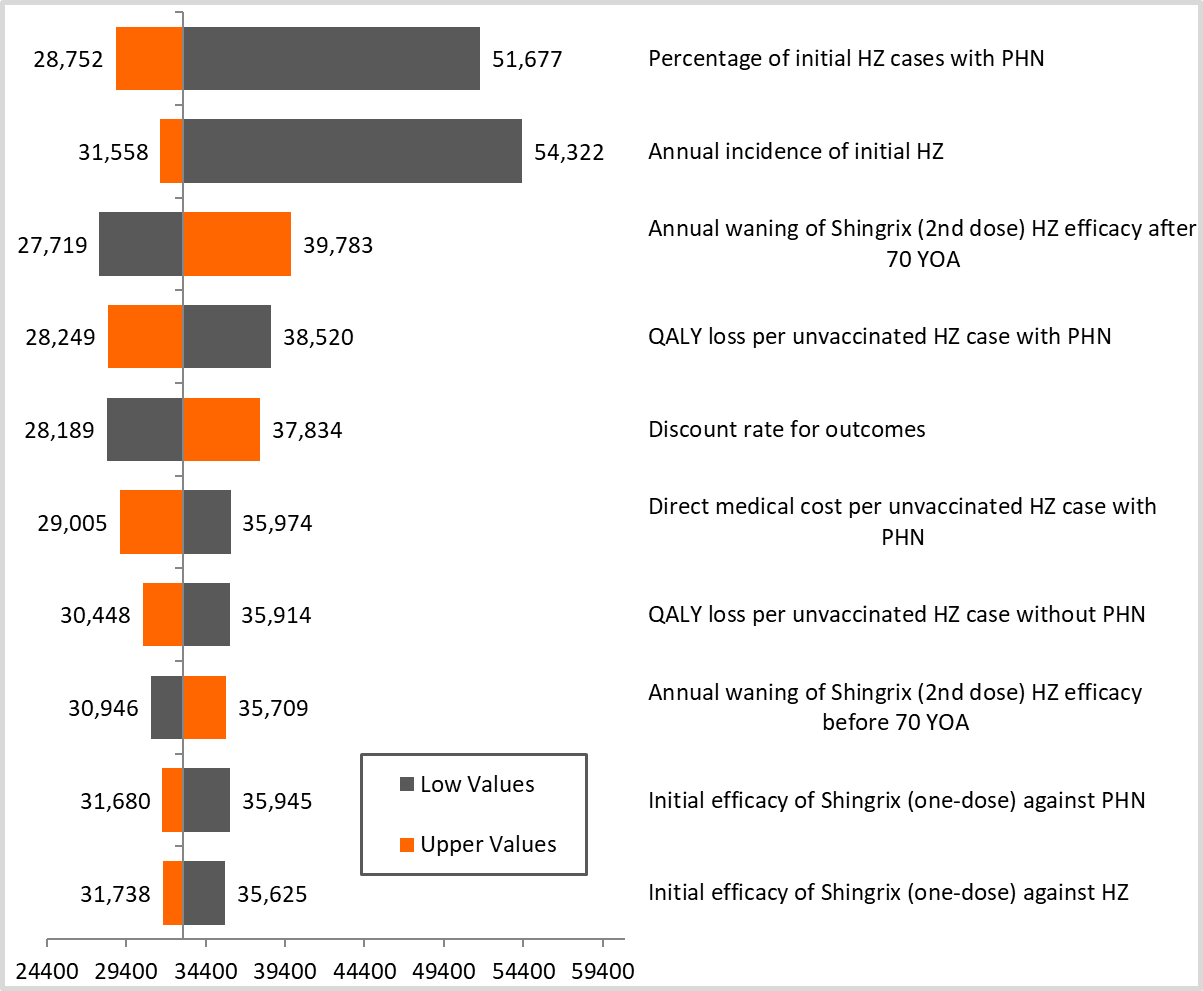


Base-case ICER: ICER of €32,956/QALY.

DSA: deterministic sensitivity analysis; HZ: herpes zoster; PHN: postherpetic neuralgia; QALY: quality-adjusted life year; RZV: adjuvanted recombinant zoster vaccine; YOA: years of age.

Figure S4: Population ≥ 50 YOA – a) PSA results for the of 1,000 Monte-Carlo simulations. b) Cost-effectiveness acceptability curve.


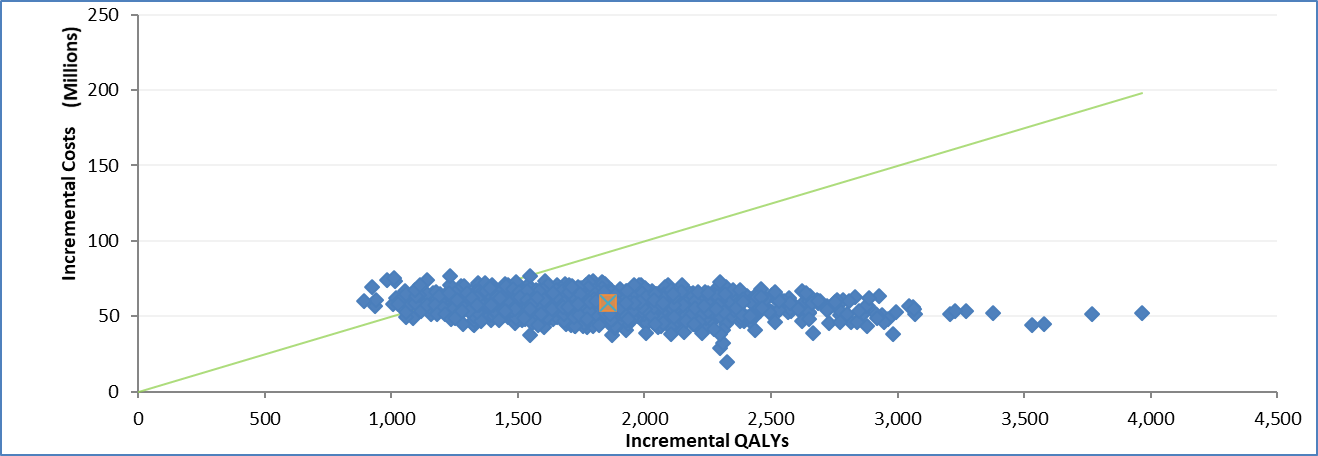


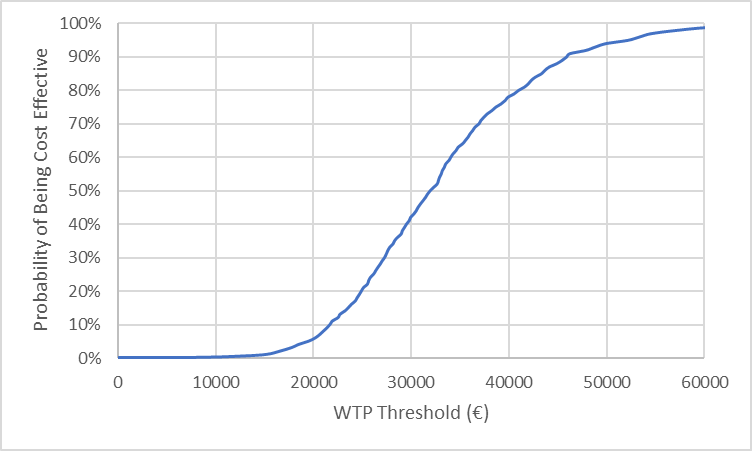


1. The orange dot is indicating the base-case ICER of €31,735/QALY, the green line presents a hypothetical WTP threshold of €50,000/QALY.
2. ICER: incremental cost-effectiveness ratio; PSA: probabilistic sensitivity analysis; QALY: quality-adjusted life year; WTP: willingness to pay; YOA: years of age.

Figure S5: Population ≥ 60 YOA – a) PSA results for the of 1,000 Monte-Carlo simulations. b) Cost-effectiveness acceptability curve.


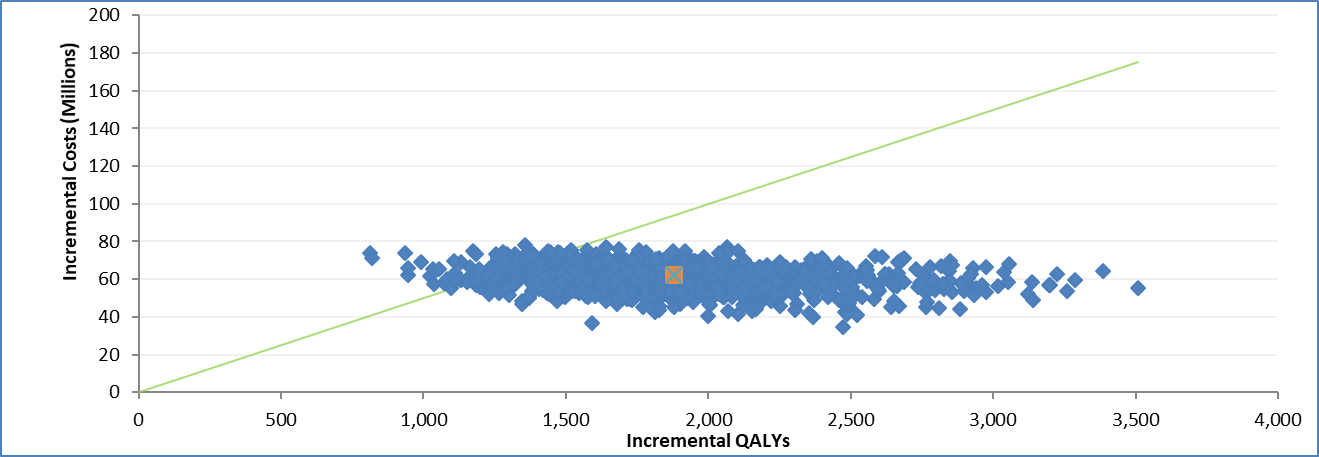


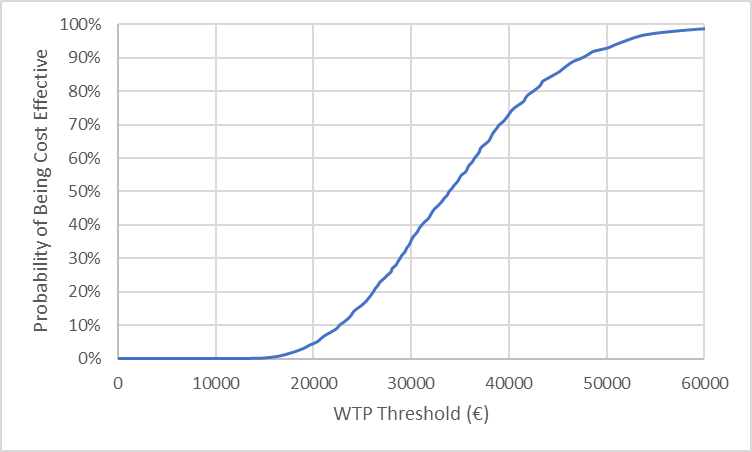


1. The orange dot is indicating the base-case ICER of €32,956/QALY, the green line presents a hypothetical WTP threshold of €50,000/QALY.
2. ICER: incremental cost-effectiveness ratio; PSA: probabilistic sensitivity analysis; QALY: quality-adjusted life year; WTP: willingness to pay; YOA: years of age.

Figure S6: Incremental cost-effectiveness Ratios (ICERs) by price per dose of RZV: direct and indirect costs updated to 2020 values


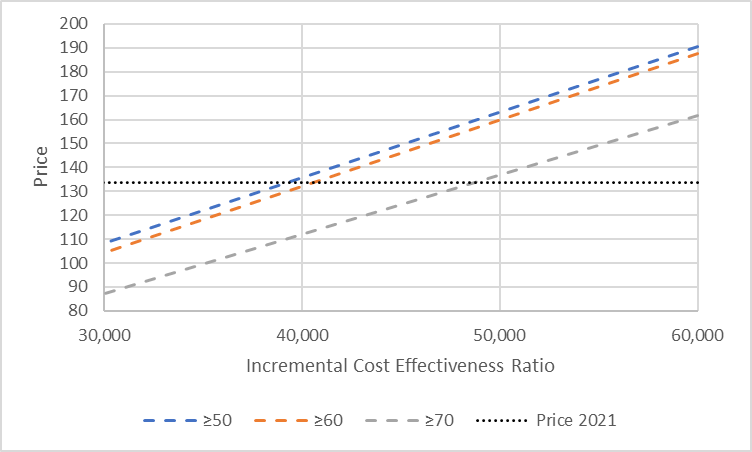


RZV: recombinant zoster vaccine; YOA: years of age.

The 2021 price of RZV is €133.62 per dose (i.e. average price to be paid by payers for one dose across all 17 health care regions via office supply) corresponding to a price to wholesaler (PTW) of €106.18 per dose.

**Table S3: Estimated annual herpes zoster cases by age group in Germany**

| **Age group** | **Population** | **Incidence** | **HZ cases** | **Percent** |
| --- | --- | --- | --- | --- |
| 50-59 YOA | 12,993,405 | 0.007716 | 100,257 | 26.6% |
| 60-64 YOA | 5,202,056 | 0.009972 | 51,875 | 13.8% |
| 65-69 YOA | 4,331,884 | 0.011371 | 49,258 | 13.1% |
| 70-79 YOA | 8,239,091 | 0.013336 | 109,877 | 29.2% |
| ≥ 80 YOA | 4,729,203 | 0.013804 | 65,282 | 17.3% |
| Total |  |  | 376,548 |  |

HZ: herpes zoster; YOA: years of age.

Incidence values as reported in Van Oorschot et al. 2019.^13^
